# Symptoms, care consumption, and healthcare costs in hospitalized patients during the first wave of the COVID-19 pandemic

**DOI:** 10.1371/journal.pone.0291548

**Published:** 2023-09-14

**Authors:** Linda Ashman Kröönström, Åsa Lundgren-Nilsson, Gunilla Kjellby-Wendt, Katharina Stibrant Sunnerhagen

**Affiliations:** 1 Institute of Neuroscience and Physiology, Section of Clinical Neuroscience, Sahlgrenska Academy, University of Gothenburg, Gothenburg, Sweden; 2 Occupational and Physical Therapy Department, Sahlgrenska University Hospital, Gothenburg, Sweden; University of Campania Luigi Vanvitelli: Universita degli Studi della Campania Luigi Vanvitelli, ITALY

## Abstract

**Background:**

We aimed to assess symptomatology post discharge from the hospital in patients with COVID-19 treated during the first wave of the COVID-19 pandemic, and to follow care consumption and healthcare costs six months post discharge.

**Methods:**

This study was a descriptive observational study over time. Data were retrieved from the Sahlgrenska University (SU) hospital registry for patients admitted to an SU hospital during March 2020 to August 2020. Of these, 1014 received a questionnaire approximately six weeks post discharge regarding symptoms. Data regarding care consumption were retrieved from the registry in the Region Västra Götaland for 529 (52.2%) patients who completed the questionnaire. Of these, 466 patients were included in the analysis of care consumption.

**Results:**

There was a reported decrease in mobility from admission to discharge in both men (p = 0.02) and women (p = 0.01). The costs of inpatient care amounted to a total of 9 601 459.20 Euro (EUR). Symptoms were reported in 436 (93.6%) patients post discharge, of which weight loss during COVID-19 was most common in both men (n = 220, 77.5%) and women (n = 107, 58.8%). During six-month follow-up, 409 (87%) patients consumed care. Of the registered visits, 868 (27.1%) were related to a COVID-19 diagnosis. The total sum of outpatient care (i.e. visits with a registered COVID-19 diagnosis) was 77 311.30 EUR.

**Conclusions:**

At discharge from the hospital, there was a decrease in mobility. Most patients had remaining sequelae post discharge. At six months post discharge, nearly 90% of patients had consumed care, with approximately 20% related to COVID-19. This indicates a persisting need for rehabilitation post discharge from hospital in patients treated for COVID-19.

## 1. Introduction

Coronavirus disease 2019 (COVID-19), caused by severe acute respiratory syndrome coronavirus 2 (SARS-CoV-2), was classified as a pandemic on March 11, 2020 by the World Health Organization (WHO). COVID-19 can impact many organs and cause a wide array of symptoms in both the acute phase of infection, as well as the post-acute phase [1,2]. The term post-COVID-19 has been identified as a condition which occurs in individuals usually 3 months from the onset [3]. A follow-up six months after hospitalization revealed that 76% of patients reported at least one symptom, with fatigue, muscle weakness, and sleep difficulties being the most common [4]. Furthermore, fatigue and muscle weakness remained the most frequently reported symptoms at one-year follow-up; however, the proportions of patients reporting symptoms was decreased [5].

Rehabilitation aims to optimize functioning and reduce disability to facilitate performing activities of daily living (ADL) and increasing autonomy, all in consideration of the patient’s own goals [6]. Symptoms with a non-specific nature, such as breathlessness or fatigue, may require clinical examination to define the impairments. Detecting such impairments is important to provide patients with the best possible rehabilitation after COVID-19 infection.

Care consumption—such as clinical examinations and rehabilitation—post discharge from the hospital in patients with COVID-19 has rarely been investigated. A US registry study showed that the burden of COVID-19 on the healthcare system was highest the first month after a COVID diagnosis and costs were driven by inpatient costs [7]. In Sweden, the healthcare is taxed financed and costs are not a reason for refraining from seeking care. A Swedish study of 158 patients showed that 44.3% had a persisting rehabilitation need at five-month follow-up [8]. Symptomatology, features, and care consumption in Swedish patients treated for COVID-19 post discharge require further evaluation. Healthcare costs in patients hospitalized due to COVID-19 regarding both inpatient and outpatient care are sparsely studied. A systematic review (including five studies from four countries of which one was a European country) regarding health care costs of hospitalized patients treated due to COVID-19, found a great heterogeneity in the reporting of health care costs [9]. Therefore, the aim of the present study was to assess the symptomatology post discharge from the hospital in patients with COVID-19 treated during the first wave of the COVID-19 pandemic and to follow care consumption and healthcare costs six months post discharge from the hospital. Additionally, we set out to assess these factors according to sex.

## 2. Study cohort and methods

### 2.1 Study cohort

This study was a descriptive observational study over time. Sahlgrenska University (SU) Hospital comprises four hospitals in Gothenburg, Sweden and serves approximately 1 million inhabitants within Greater Gothenburg. These hospitals provide outpatient as well as inpatient care, with approximately 2000 hospital beds. Patients aged ≥18, with a COVID-19 diagnosis, hospitalized (defined as at least one day) in any inpatient ward at an SU Hospital from March 2020 to August 2020 (the first wave of the COVID-19 pandemic in Sweden), were eligible for study inclusion. Patients received a questionnaire approximately six weeks post discharge from the hospital, via post or the following webpage: www.1177.se in order evaluate need for contact with the health care post-discharge. Participants were excluded from the analysis of care consumption when not discharged to own home owing to difficulties in assessing care consumption in homes were care and rehabilitation were provided in situ. As stated in the Personal Data Act (Swedish law No. SFS 1998:204), informed consent is not obligatory in order to collect data for quality registers (such as the SU-COVID registry), but there is an opt-out option. Therefore, since the data was from a quality register the requirement of informed consent was not needed. This study was approved by the Swedish Ethical Review Authority (No. 2020–03604, 2021–02028) and was performed in accordance with principles of the Declaration of Helsinki. The study followed the Strengthening the Reporting of Observational Studies in Epidemiology (STROBE) reporting guideline for observational studies [10].

### 2.2 Questionnaire

The questionnaire was designed by the management team for rehabilitation after COVID-19 (consisting of medical doctors, occupational therapists, physiotherapists, psychologists, dieticians and speech and language therapists) at SU Hospital with the aim of triaging patients`long-term follow-up of symptoms and need for care after discharge from the hospital; patients’ views were incorporated in the design. The first questionnaires were sent on July 2, 2020. The questionnaire included in total 18 questions, of which 11 were influenced by the post-stroke checklist [11] (which covers different areas of the International classification of functioning, disability and health [12]) and has been assessed regarding feasibility and relevancy in patients with stroke [13]. The questionnaire included 16 questions regarding symptoms, as follows: ADL, mobilization or walking outdoors, new sensitivity/pain, gastro-intestinal problems, worries/anxiety, cognitive difficulties such as concentration/memory affecting daily living, eating/drinking, weight loss during the hospital stay, weight loss after discharge or last follow-up, sleep, nightmares, tracheostomy, hoarseness/impact on the voice, breathing, persisting cough, and pressure wounds. The questionnaire also queried whether patients had returned to work, their studies, or a previous occupation, and the patients’ need to revisit the hospital, healthcare, or rehabilitation after discharge from the hospital (see S1 Table in S1 File). The questionnaire was available in Swedish on the webpage. Patients who did not use the web-page, or patients whose native language was not Swedish received a questionnaire by post. The questionnaire was translated by the region’s professional translators into five other languages (Arabic, English, Persian, Serbo-Croatian, and Somali), commonly used by patients in the catchment area.

### 2.3 Data sources

Data for patients treated in an SU hospital for COVID-19 infection were retrieved from the SU´s hospitals’ own registry (SU-COVID registry). This registry was launched in 2020 and is held by Region Västra Götaland, SU Hospital and administered by Gothia Forum, Gothenburg, Sweden. Data from the SU-COVID registry includes information regarding functional capabilities prior to hospitalization, inpatient care, and discharge data. Data were retrieved from the registry in the Region Västra Götaland (Vega registry) for inpatient and outpatient care as well as care consumption six months after hospital discharge. The Vega registry was launched in 2000 and contains data for both public and private care within the region.

### 2.4 Definitions of central concepts

Co-morbidities were defined according to the updated Charlson comorbidity index (CCI) where a sum is created to predict 1-year mortality [14]. We used the term care consumption to include all visits to outpatient hospital and primary care such as clinical examinations and rehabilitation post discharge from the hospital. The following seven codes from the International Classifications of Diseases Tenth Revision (ICD-10) were used to identify a COVID-19 diagnosis: B34.2 (coronavirus infection, unspecified location) (in use according to a decision from the WHO from 2020-02-03), Z86.1A (COVID-19 in patient’s own medical history) (in use from 2020-06-01), U071 (COVID-19, virus identified), U072 (COVID-19, virus not identified), U089 (personal history of COVID-19, unspecified), U099 (post COVID-19 condition, unspecified), and U109 (multisystem inflammatory syndrome associated with COVID-19, unspecified) (all in use from 2021-01-01). Regarding interventions, the following ICD-10 code was used for analysis: ZV100 (intervention related to COVID-19). In the analysis of the costs of care consumption for inpatient care, we used the Swedish standard sum of costs per patient for 2021, calculated by the Vega registry for patients with ICD-10 codes U071 and U072. The sum includes all costs during inpatient care such as care in an intensive care unit (ICU), staff, facilities, technical equipment, laboratory tests, and medications during hospitalization. The costs for individuals aged 18–65 years was 16 717.70 Euro (EUR), and 21 255.40 EUR for those aged ≥66 years [15]. For outpatient care, we used the following standard sum for doctors’ visits of 151.40 EUR, and 61.70 EUR for visits to all other healthcare professionals. Calculated values were converted from Swedish Krona (SEK) to EUR based on conversion rates on January 23, 2023.

### 2.5 Statistical methods

Data were analysed using IBM Statistical Package for Social Sciences (SPSS) 28.0 and 29.0 (IBM Corp., Armonk, NY, USA). The CCI was calculated using the calculator published by Prommik et al. [16]. Demographic data are presented as means (standard deviation [SD]), median [interquartile range, (IQR)] or [min, max]. Tests between two independent groups were carried out using the parametric Student *t*-test, or if non-normally distributed, a nonparametric Mann–Whitney U test. Comparisons between two dependent groups were done using the Wilcoxon signed rank test. All tests were two-sided and *p* <0.05 was considered to represent statistical significance.

The questionnaire was treated as an ordinal scale and results presented as number and (%). Regarding analysis of care consumption, we excluded visits that were not healthcare related. Diagnoses were listed in hierarchical order, with the first being the most important as there could be multiple diagnoses for each visit. Therefore, when applicable, we chose the first diagnosis for analysis.

## 3. Results

### 3.1 Patient recruitment

**Fig 1:**
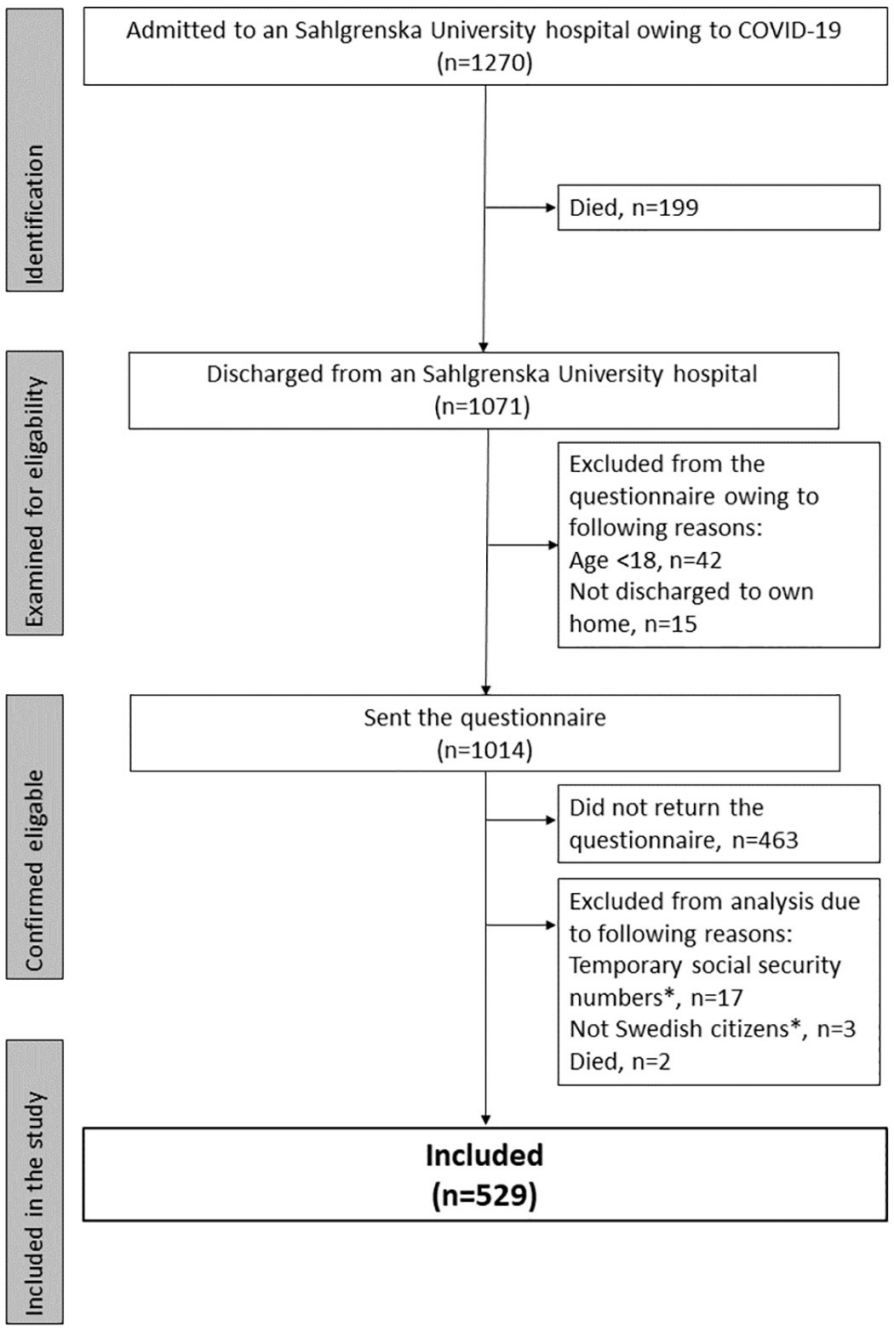
Patient recruitment diagram. COVID-19, coronavirus disease 2019.

In total, 1270 patients with a COVID-19 diagnosis were admitted to an SU hospital during the first wave of the COVID-19 pandemic. Of these, 199 (15.7%) patients died. In total, 1071 patients were discharged from hospital, of which 42 (3.9%) were under age 18 years. Fifteen (1.4%) patients who were not discharged home were not sent the questionnaire owing to difficulties in assessing care consumption. Of the 1014 patients who were sent the questionnaire, 463 (45.7%) did not return the questionnaire, 3 (0.3%) were not Swedish citizens, and 17 (1.7%) had temporary social security numbers. Five hundred and twenty-nine (52.2%) patients responded to the questionnaire and their data were retrieved from the Vega registry. A flowchart of patient recruitment is presented in Fig 1.

Questionnaires where the time from discharge to survey completion was ≤28 or ≥135 days were treated as having internal missing data (n = 61, 11.5%). Furthermore, results were excluded if the questionnaire was insufficiently complete or included contradictory responses (n = 2, 0.03%).

### 3.2 Characteristics of the study population at admission and inpatient care

Of the 529 included patients, the mean age was 58.77 (15.1) years, with a predominance of male patients responding to the questionnaire (n = 327, 61.8%) (Table 1). In total, 515 (97.4%) patients responded to the Swedish version of the questionnaire, 9 (1.7%) responded in Arabic, 0 (0%) in English language, 2 (0.4%) in Persian, 1 (0.2%) in Serbo-Croatian, and 2 (0.4%) completed the Somali version of the questionnaire.

**Table 1:** Characteristics of the included patients, N = 529.

	Characteristic	All patients n = 529	Men n = 327	Women n = 202
**At admission**	Age (years), mean (SD)	58.7 (15.1)	58.3 (14.4)	59.6 (16.3)
	Living alone, n (%)	124 (23.4)	64 (19.6)	60 (29.7)
	BMI (kg/m^2^), median [IQR]*	n = 210 28.3 [25.0, 31.8]	n = 137 28.0 [25.0, 31.2]	n = 73 28.7 [24.4, 33.2]
	CCI, median [min, max]	0.0 [0, 6]	0.0 [0, 6]	0.0 [0, 4]
	0 points, n (%)	418 (79.0)	269 (82.3)	149 (73.8)
	1–2 points, n (%)	105 (19.8)	54 (16.5)	51 (25.2)
	3–4 points, n (%)	5 (0.9)	3 (0.9)	2 (1.0)
	≥5 points, n (%)	1 (0.2)	1 (0.3)	0 (0.0)
**Inpatient care**	Requiring care in an ICU, n (%)	71 (13.4)	53 (16.2)	18 (8.9)
	Mechanical ventilation, n (%)	61 (11.5)	46 (14.1)	15 (7.4)
	Supplemental oxygen, n (%)	394 (74.5)	252 (77.1)	142 (70.3)
	High-flow nasal cannula, n (%)	118 (22.3)	83 (25.4)	35 (17.3)
	Difficulties with oral nutrition, n (%)	131 (24.8)	75 (22.9)	56 (27.7)
	Pressure wounds, n (%)	21 (4.0)	18 (5.5)	3 (1.5)
	Physiotherapy, n (%)	157 (29.7)	100 (30.6)	57 (28.2)
	Occupational therapy, n (%)	126 (23.8)	82 (25.1)	44 (21.8)
**At discharge**	BMI (kg/m^2^), median [IQR]*	n = 60 28.0 [23.7, 30.5]	n = 41 27.4 [23.4, 30.1]	n = 19 28.7 [25.0, 33.8]
	Length of hospital stay, days			
	mean (SD)	9.8 (15.8)	11.1 (17.3)	7.6 (12.8)
	median [IQR]	5.0 [2.0, 10.0]	5.0 [3.0, 11.0]	5.0 [2.0, 9.0]
	Readmission to hospital for COVID-19 within 7 days, n (%)	16 (3.0)	8 (2.4)	8 (4.0)
	Readmission to hospital for COVID-19 within 2 months, n (%)	18 (3.4)	9 (2.8)	9 (4.5)

SD, standard deviation; BMI, body mass index; IQR, inter quartile range; CCI, Charlson comorbidity index; ICU, intensive care unit; COVID-19, coronavirus disease 2019.

*BMI values were excluded if not assessed one week post or prior to admission (n = 47) or discharge (n = 6).

Seventy-one (13.4%) patients required care in an ICU (Table 1). The proportion of men who were dependent (that is, requiring help with toilette visits and/or dressing/undressing) at the time of admission to hospital was 8 (2.4%), which increased to 23 (7.0%) (p<0.001) at discharge; and the equivalent numbers in women were 9 (4.5%) versus 23 (11.4%) (p<0.001) (Table 2). Furthermore, there was a reported decrease in mobility from admission to discharge in both men (p = 0.02) and women (p = 0.01). The costs of inpatient care for patients aged 18–65 years (n = 362) was 6 051 807.40 EUR, and that for patients aged ≥66 (n = 167) was 3 549 651.80 EUR, with a total estimated sum for inpatient care of 9 601 459.20 EUR.

**Table 2:** Functional capabilities on admission and at discharge, N = 529.

Characteristic	On admission		At discharge		p-Value*	p-Value*
	Men n = 327	Women n = 202	Men n = 327	Women n = 202	Men	Women
**Patient’s living situation**					0.89	0.76
Without care	303 (92.7)	172 (85.1)	290 (88.7)	162 (80.2)		
Home care	9 (2.8)	16 (7.9)	23 (7.0)	27 (13.4)		
Healthcare	1 (0.3)	0 (0.0)	4 (1.2)	2 (1.0)		
Home and healthcare	6 (1.8)	5 (2.5)	10 (3.1)	11 (5.4)		
**P-ADL dependent** **	8 (2.4)	9 (4.5)	23 (7.0)	23 (11.4)	<0.001	<0.001
**Mobility**	n = 322	n = 202	n = 322	n = 202	0.02	0.01
Walks independently in-/outdoors	308 (94.2)	180 (89.1)	286 (87.5)	165 (81.7)		
Dependent, in-/outdoors	2 (0.6)	2 (1.0)	10 (3.1)	5 (2.5)		
Dependent walking outdoors	3 (0.9)	5 (2.5)	4 (1.2)	7 (3.5)		
Independent with rollator	7 (2.1)	10 (5.0)	16 (4.9)	15 (7.4)		
Dependent with rollator	1 (0.3)	2 (1.0)	4 (1.2)	7 (3.5)		
Wheelchair dependent	1 (0.3)	3 (1.5)	2 (0.6)	3 (1.5)		

Data are n (%). P-ADL, personal activities of daily living.

* Wilcoxon signed rank test was used for the analysis.

** Patients requiring help with toilette visits and/or dressing/undressing.

### 3.3 Symptoms and features post discharge from hospital

**Fig 2:**
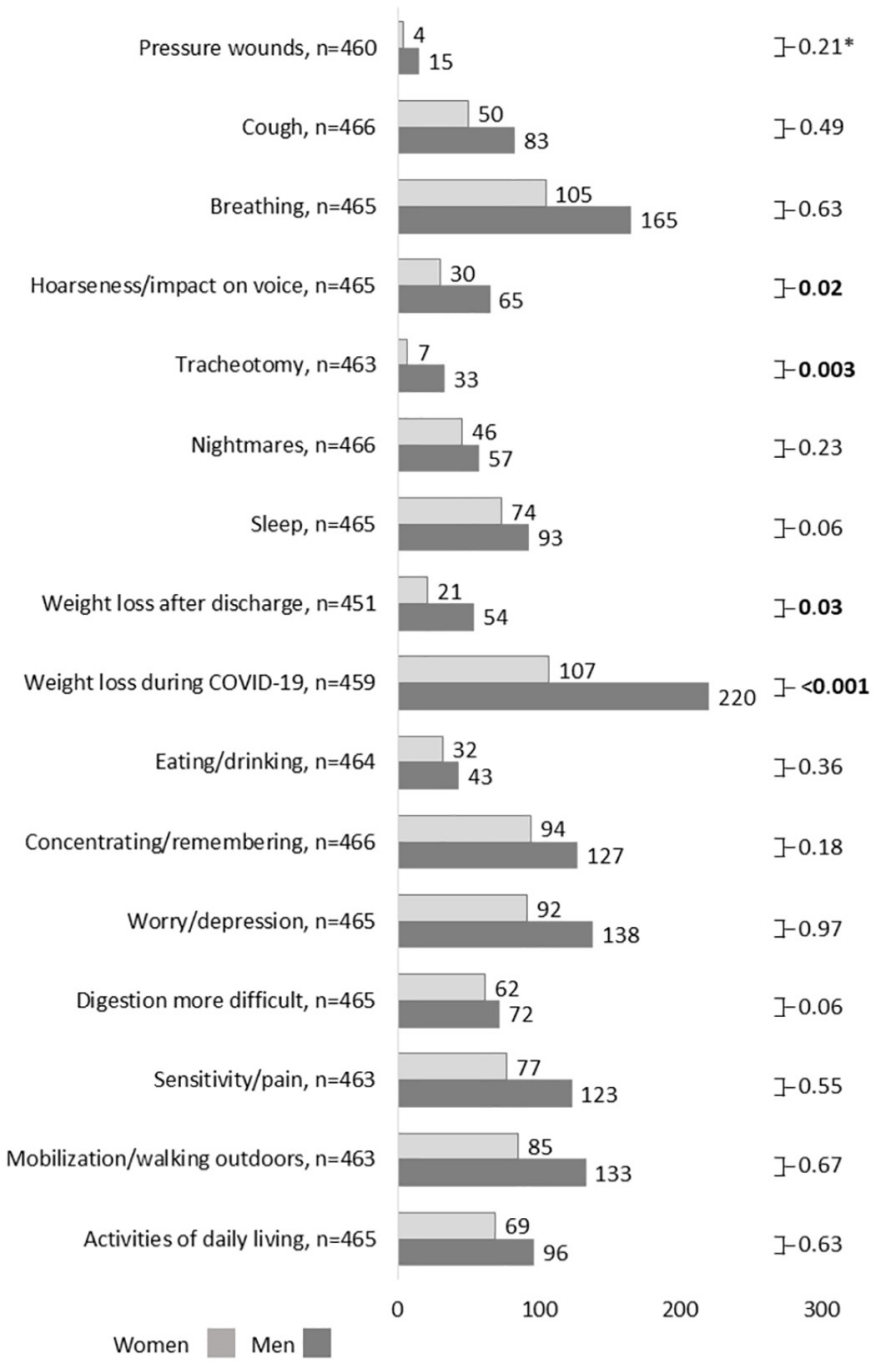
Men (n = 284) and women (n = 182) reporting symptoms post discharge after hospitalization owing to COVID-19, N = 466. COVID-19, coronavirus disease 2019. Bold figures denote statistical significance.

Four hundred and sixty-six (88.1%) patients answered the questionnaire at ≥28 to ≤135 days post hospital discharge and were included in the analysis of symptomatology and care consumption. Of these, 284 (60.1%) were male patients. The mean length of time between discharge and completing the questionnaire was 69.6 (±23.0) days. Four hundred and thirty-six (93.6%) patients reported symptoms post discharge, with a mean number of symptoms of 5.3 (3.6%) (out of 16 possible). The number of women who reported symptoms was 168 (92.3%) versus 268 (94.4%) men (p = 0.42). Two hundred and forty-three (52.1%) patients reported being back at work, their studies, or a previous occupation whereas 54 (11.6%) patients reported being on sick leave; 23 (4.9%) were unemployed, and 131 (28.1%) were not working age. The most frequently reported symptom post discharge was weight loss during COVID-19 infection for both men (n = 220, 77.5%), and women (n = 107, 58.8%) (Fig 2); symptom severity is displayed in S1 Table in S1 File. The proportion of men who had a tracheotomy was significantly greater than the proportion of women (p = 0.003), and men reported hoarseness (p = 0.02), weight loss during COVID-19 infection (p<0.001), and weight loss after discharge (p = 0.03) significantly more often than women.

### 3.4 Care consumption post discharge from hospital

Of the 466 patients who were included in the analysis of care consumption, 409 (87%) used care (Table 3). Of the registered visits, 868 (27.1%) involved a COVID-19 diagnosis, among which 487 (56.1%) visits were to a physio-therapist. The total sum of costs for outpatient care (i.e., visits with a registered COVID-19 diagnosis) amounted to 77 311.30 EUR.

**Table 3:** Outpatient care consumption and healthcare costs in patients hospitalized during six month follow-up, N = 466.

	Characteristic	All patients n = 466	Men n = 284	Women n = 182	p-Value	Cost in EUR*
	Number of patients who consumed care, n (%)	409 (87.8)	245 (86.3)	164 (90.1)	1.00^a^	
	Total number of visits	n = 3536	n = 2347	n = 1189	<0.001^b^	
	Number of visits, mean (SD)	8.7 (11.8)	9.7 (13.5)	7.3 (8.5)	<0.001^b^	
	Number of visits, median [IQR]	4 [2.0, 10.0]	5.0 [2.0, 11.0]	4.0 [2.0, 8.0]	0.95^a^	
	Multiple/coordinated visits same day, n (%)	221 (6.3)	134 (5.7)	87 (7.3)	0.06^a^	
	Time between discharge and first appointment (days), mean (SD)	38.6 (42.4)	37.8 (42.2)	39.8 (42.9)	0.89^b^	
	Time between discharge and first appointment (days), median [IQR]	22.0 [7.0, 55.5]	21.0 [7.0, 53.5]	26.0 [7.2, 57.7]	0.45^a^	
**Diagnoses**	No. of appointments with an ICD-10 diagnosis code	n = 3202	n = 2128	n = 1074		
	COVID-diagnosis, n (%)	868 (27.1)	648 (30.4)	220 (20.5)	<0.001^b^	
	No. of first appointments with an ICD-10 diagnosis code, n	n = 370	n = 220	n = 150		
	Primary COVID-diagnosis in the first appointment, n (%)	72 (19.4)	46 (20.9)	26 (17.3)	0.68^a^	
**Appointment**	No. of appointments with an intervention code	n = 2218	n = 1537	n = 681		
	Appointments with a COVID-19 intervention, n (%)	314 (14.1)	273 (17.8)	41 (6.0)	1.00^a^	
**Professionals**	Different professionals regarding all visits with a registered COVID-diagnoses, n (%)	(n = 868)	(n = 648)	(n = 220)		
	Doctor	265 (30.5)	162 (25.0)	103 (46.8)	<0.001^a^	40 110.80
	Occupational therapist	75 (8.6)	60 (9.3)	15 (6.8)		37 200.50**
	Physiotherapist	487 (56.1)	402 (62.0)	85 (38.6)		
	Nurse/assistant nurse	36 (4.1)	22 (3.4)	14 (6.4)		
	Other	5 (0.6)	2 (0.3)	3 (1.4)		

EUR, Euro; COVID-19, coronavirus disease 2019; SD, standard deviation; IQR, inter quartile range; ICD-10, International Classification of Diseases Tenth Revision.

^a^Mann–Whitney U test.

^b^Student *t* -test.

* Based on currency exchange rates as of January 23, 2023.

**Costs calculated for the total of all visits to all other healthcare professionals.

Analysis of patients who consumed care (n = 409) as opposed to patients who did not (n = 57), showed that patients who used care had longer hospital stays (9.6±14.3 days vs. 4.2±3.6 days, p<0.001) and higher CCI scores (0.35±0.7 vs. 0.05±0.2, p<0.001). However, we found no difference in the proportions regarding sex (men: n = 245, 59.9% vs. n = 39, 68.4%, p = 0.22) or admittance to an ICU (n = 51, 12.5% vs. n = 4, 7.0%, p = 0.23). Furthermore, there was no significant difference regarding the mean patient age (60.2±15.3 years vs. 52.7±14.3 years, p = 0.28) between these groups.

## 4. Discussion

We report our findings regarding symptomatology, care consumption and costs post discharge from the hospital in patients treated during the first wave of the COVID-19 pandemic. Most patients (n = 436, 93.6%) reported symptoms post discharge from the hospital, and the mean number of symptoms was 5.3 (3.6%). These results are in accordance with other studies covering a wide variety of persisting symptoms after COVID-19 [1,2]. The results of the present study showed that the most frequently reported symptom post discharge was weight loss during COVID-19 in both men (77.5%), and women (58.8%). A previous study in hospitalized patients during the first COVID-19 wave reported that the most common symptom 4–8 weeks post discharge was fatigue, which was reported in 72% of patients treated in an ICU and in 60.3% of patients treated in other wards [17]. Morin et al. [18] reported that 51% of patients reported symptoms 4 months post discharge, and that fatigue was the most common symptom (31.1%). A Swedish study with a 4-month follow-up also reported fatigue (38%) and limb weakness (31%) as the most common symptoms, and the number of patients reporting these symptoms increased with increasing severity of COVID-19 [19].

Studies reporting healthcare costs of inpatient as well as outpatient care in hospitalized patients during COVID-19 are sparse. In the present study, the total sum of inpatient care amounted to 9 601 459.20 EUR. Costs per patient were higher in older patients (≥66 years) than the costs of younger patients (18–65 years), explained by the fact that older people are often more severely ill with a viral infection and may require more inpatient care as well as care in an ICU compared with younger patients. A previous study from Saudi Arabia also found a higher cost for patients aged >60 years as opposed to younger patients [20]. The same study showed that costs were higher for patients who were critically ill with COVID-19 infection, in comparison with patients who had milder illness, and care costs for patients with prior comorbidities were higher than those for patients with no comorbidities [20].

The severity of COVID-19 can be categorized according to the WHO Clinical Progression Scale [21]. Patients requiring ICU care may represent a special group because these patients are assumed to have more severe infection and may therefore present other or more pronounced symptoms. In the present study, 71 patients (13.4%) required care in an ICU. Other researchers have estimated that approximately 20% of patients hospitalized with COVID-19 exhibit symptoms requiring ICU care [22]. The consequences for patients not specifically treated for COVID-19 but treated in an ICU have been previously assessed. Patients lose as much as 20% of muscle mass during the first 10 days of ICU care, which may be the result of many contributing factors such as critical illness, muscle unloading, comorbidities, or ICU treatments [23]. Most patients in the present study required supplemental oxygen (n = 394, 74.5%); these results are in agreement with those of other studies reporting that supplemental oxygen therapy is required in more than 75% of hospitalized patients with COVID-19 [22]. Furthermore, 61 patients (11.5%) required mechanical ventilation, which indicates greater disease severity according to the WHO Clinical Progression Scale [21].

After being hospitalized owing to COVID-19, there was an increased proportion of both men (p<0.001) and women (p<0.001) who required help with personal ADL. A meta-analysis in adults aged ≥65 years reported decreased independence in approximately 30% of hospitalized patients [24]. Similarly, at discharge, the number of men and women who had decreased mobility was increased in both men (p = 0.02) and women (p = 0.01). A previous study found that 7.6% of patients received walking aids after COVID-19, which they still needed five months post discharge [8]. Muscle weakness and fatigue are reported to be the most common symptoms at six month follow-up in hospitalized patients with COVID-19 [4], and muscle weakness may negatively impact walking ability. Older patients and patients with comorbidities may be more vulnerable to the effects of severe illness, and rehabilitation is beneficial for them to maintain or regain a level of independence [25]. In addition to the specific disease or pathogen, immobilization and other factors during hospitalization contribute to the increased need for care post discharge.

We also aimed to analyse care consumption post discharge from the hospital and found a clear preponderance of patients who sought outpatient care (n = 409, 87.8%). The costs of outpatient care (i.e., visits with a COVID-19 diagnosis) during the 6 months following discharge from the hospital amounted to 77 311.30 EUR. A large US-based registry study with a six month follow-up showed that the burden of COVID-19 on the healthcare system was highest in the month after diagnosis [7]. Comorbidities prior to COVID-19 could be one reason for seeking care. Our results showed that 111 (20.9%) patients had comorbidities prior to COVID-19, and patients who consumed care had a higher CCI score than patients who did not consume care (p<0.001). Patients with comorbidities, as well as those with other health issues, may already have ongoing medical needs or rehabilitation requiring outpatient care. Furthermore, patients who consumed care had a longer hospital stay than those who did not consume care (p<0.001). Of all visits, 27.1% were registered with an ICD-10 COVID-19 diagnosis. These results are somewhat higher than those from a previous study showing that 18% of patients had visited an outpatient clinic owing to COVID-19 at one year after hospitalization [5]. Furthermore, we found that a physio-therapist (n = 487, 56.1%) was the most commonly sought health professional. This may be explained by the nature of physiotherapy interventions, which often include exercise training involving repeated visits. The results of the present study therefore confirm the findings of previous studies regarding the need for rehabilitation post discharge from hospital in patients treated for COVID-19. Rehabilitation needs have previously been assessed in a Swedish context using the Rehabilitation Complexity Scale–Extended, which showed that 44.3% of patients had persisting rehabilitation needs five4 months post discharge from the hospital [8].

There is a natural course of recovery after a viral infection; however, assessing signs and symptoms is a way of planning and providing the best available individualized rehabilitation for patients treated for COVID-19. Rehabilitation is essential during all phases in management of the COVID-19 pandemic [26]. An increasing number of patients will require rehabilitation owing to COVID-19, and the healthcare system must address these needs. It is important to detect the main focus for each patient to provide adequate rehabilitation. Multidisciplinary teams may be beneficial in caring for patients with COVID-19 who have a wide array of persisting symptoms [27] that may be both physical and cognitive [1]. Prolonged work absence is costly and rehabilitation may help shorten the time to return to a work. Results of the present study showed that 52.1% of patients reported being back at work, their studies, or a previous occupation whereas 54 (11.6%) patients reported being on sick leave at the time of completing the questionnaire. A previous Swedish study reported that sick leave owing to COVID-19 is more common in individuals needing inpatient care, those with prior sick leave, and older people [28]. Furthermore, recurrent sick leave owing to COVID-19 is more common in older individuals, women, and people who had been on sick leave prior to the COVID-19 pandemic [29].

There was a predominance of male patients admitted to the hospital, perhaps explaining why more men (n = 284, 60.1%) responded to the questionnaire. Previous articles have also reported that more men than women were hospitalized during the first wave of the COVID-19 pandemic [30]. There was a greater proportion of men than women who reported having had a tracheotomy (p = 0.003), which may have entailed subsequent hoarseness; this has also been reported in a larger proportion of men than women (p = 0.02). The most frequently reported symptom post discharge was weight loss during COVID-19 infection, and weight loss both during and after COVID-19 was reported by a greater proportion of men than women (p<0.001; and p = 0.03 respectively).

COVID-19 is a newly emerged pandemic with unknown impacts and long-term effects; therefore, following symtomatology and care consumption over a longer period is important. Treatment, medications, and the degree of vaccination may have altered subsequent COVID-19 waves after the first and may continue to do so in future epidemic waves. In England, for example, there has been a substantial improvement in survival among patients admitted to critical care from March to April 2020, in comparison with May–June 2020 [31]. Therefore, further studies are required to investigate differences in COVID-19 waves regarding post discharge symptoms, care consumption, and the need for rehabilitation.

### 4.1 Strengths and limitations

This study was not primarily a research project but rather a project in a clinical setting aiming to assess symptoms post COVID-19. Strengths of the present study include the registration of all (to our best knowledge) patients hospitalized for COVID-19 in our hospital, serving approximately 1 million inhabitants. The novelty of the topic regarding calculations of costs for inpatient and outpatient care is another strength, despite being derived from estimates. We also recognize the following limitations. The response rate was 52.2% and since the aim was to reach those that perceived a need of care post-discharge, this level seems acceptable. No reminders were sent out due to the work load during the pandemic and the fact that patients answered the questionnaire when they felt the need for help. Therefore, the time span within which we included questionnaire responses was broad. Furthermore, the broad time span is explained by the fact that the questionnaire was first sent out on July 2 and aimed to include the first patients hospitalized with COVID-19 infection in March in Sweden. Patients with persisting symptoms, sequelae, and greater needs for rehabilitation may have had a higher degree of motivation to complete the questionnaire as the sum of the scores resulted in a follow-up. If the questionnaire had been available in more languages, this would have potentially increased the number of patients responding to the questionnaire. Data were retrieved from registries and patients may have been unaware that their data were kept in a registry; however, the data are presented at group level and therefore, no individual data can be identified. The questionnaire was influenced by the post-stroke checklist [11], which assessed remining needs in different domains after hospital discharge, owing to no COVID-19-specific questionnaires being available when the questionnaire was created. Therefore, there is a risk of missing symptoms, such as fatigue, which is evidently common in patients after COVID-19 infection. We tried to analyse and group the type of intervention patients received at outpatient visits; however, this was impossible because the interventions were very non-specific. Furthermore, not all outpatient appointments had a registered diagnosis or an ICD-10 intervention code; thus, there may be under-reporting of COVID-19-related visits. Finally, because data were retrospectively collected, it was not possible to retrieve all desired data. Missing data was present for the BMI variable both at admission and at discharge due to not being registered in the patients’ medical charts, but would have been of great interest in the present study especially as the symptom “weight loss during COVID-19 infection” was the most commonly reported symptom.

## 5. Conclusions

At discharge from the hospital, patients reported a decrease in mobility, and the proportion of patients who required help with personal-ADL was increased. Most patients had remaining sequelae post discharge. At six months post discharge, nearly 90% of patients had used care, among which approximately 20% were related to COVID-19. This indicates a persisting need for rehabilitation and care consumption post discharge from the hospital among patients treated for COVID-19.

## Supporting information

S1 Checklist STROBE Statement—Checklist of items that should be included in reports of observational studies. (DOCX)

S1 File Symptomatology, return to previous occupation and if the patient had sought care post discharge from hospitalization due to COVID-19, N = 466. (DOCX)
