# Supplementary material for: Symptoms, care consumption, and healthcare costs in hospitalized patients during the first wave of the COVID-19 pandemic
Source: PLoS One. 2023 Sep 14;18(9):e0291548. doi: 10.1371/journal.pone.0291548 (PMC10501636; doi:10.1371/journal.pone.0291548)
Supplement: S1 File — (DOCX) [file pone.0291548.s002.docx]

**Supporting information**

1. **Results**

The results regarding symptomatology, return to previous occupation and if the patient had sought care, are presented in Supporting information, Table 1.

**Supporting information, Table 1.** Symptomatology, return to previous occupation and if the patient had sought care post discharge from hospitalization due to COVID-19, N=466

| Question | Possible answer | Men  n=284 | Women  n=182 |
| --- | --- | --- | --- |
| **1.** Do you think it is more difficult to manage everyday activities | No | 188 (66.2) | 112 (61.5) |
| after COVID-19? (n=465) | Yes, slightly more difficult | 49 (17.3) | 36 (19.8) |
|  | Yes, more difficult | 35 (12.3) | 22 (12.1) |
|  | Yes, a lot more difficult | 12 (4.2) | 11 (6.0) |
| **2.** Do you think it is more difficult to walk or move around outdoors | No | 150 (52.8) | 95 (52.2) |
| after COVID-19? (n=463) | Yes, slightly more difficult | 63 (22.2) | 39 (21.4) |
|  | Yes, more difficult | 54 (19.0) | 31 (17.0) |
|  | Yes, a lot more difficult | 16 (5.6) | 15 (8.2) |
| **3.** Do you have any new pains or sensory problems after COVID-19? | No | 160 (53.3) | 103 (56.6) |
| (n=463) | Slight | 48 (16.9) | 42 (23.1) |
|  | Some | 57 (20.1) | 26 (14.3) |
|  | A lot | 18 (6.3) | 9 (4.9) |
| **4.** Do you feel that digestion is more difficult after COVID-19? | No | 212 (74.6) | 119 (65.4) |
| (n=465) | Yes, slightly more difficult | 29 (10.2) | 27 (14.8) |
|  | Yes, more difficult | 35 (12.3) | 27 (14.8) |
|  | Yes, a lot more difficult | 8 (2.8) | 8 (4.8) |
| **5.** Do you have difficulties with worry or depression after COVID-19? | No | 146 (51.4) | 89 (48.9) |
| (n=465) | Yes, slightly more difficult | 56 (19.7) | 38 (20.9) |
|  | Yes, more difficult | 56 (19.7) | 38 (20.9) |
|  | Yes, a lot more difficult | 26 (9.2) | 16 (8.8) |
| **6.** Do you find it more difficult to think, concentrate or remember | No | 157 (55.3) | 88 (48.4) |
| things that affect your everyday life after COVID-19? (n=466) | Yes, slightly more difficult | 38 (13.4) | 36 (19.8) |
|  | Yes, more difficult | 67 (23.6) | 35 (19.2) |
|  | Yes, a lot more difficult | 22 (7.7) | 23 (12.6) |
| **7.** Do you find it more difficult to eat or drink after COVID-19? | No | 241 (84.9) | 148 (81.3) |
| (n=464) | Yes, slightly more difficult | 13 (4.6) | 14 (7.7) |
|  | Yes, more difficult | 24 (8.5) | 18 (9.9) |
|  | Yes, a lot more difficult | 6 (2.1) | 0 (0) |
| **8.** Have you lost weight during your COVID-19 infection? (n=459) | No | 62 (21.8) | 70 (38.5) |
|  | Yes | 220 (77.5) | 107 (58.8) |
| **9.** Have you lost weight since coming home from hospital or since your | No | 223 (78.5) | 153 (84.1) |
| last check-up? (n=451) | Yes | 54 (19.0) | 21 (11.5) |
| **10.** Do you find it difficult to sleep after COVID-19? (n=465) | No | 190 (66.9) | 108 (59.3) |
|  | Yes | 93 (32.7) | 74 (40.7) |
| **11.** Have you had nightmares after COVID-19? (n=466) | No | 227 (79.9) | 136 (74.7) |
|  | Yes | 57 (20.1) | 46 (25.3) |
| **12.** Did you have a tracheotomy (an opening in your throat)? | No | 250 (88.0) | 173 (95.1) |
| (n=463) | Yes, the hole has now healed and I can talk as usual | 21 (7.4) | 4 (2.2) |
|  | Yes, but I have problems after the tracheotomy | 12 (4.2) | 3 (1.6) |
| **13.** Are you hoarse or do you have any other problems with your | No | 218 (76.8) | 152 (83.5) |
| voice after COVID-19? (n=465) | Yes | 65 (22.9) | 30 (16.5) |
| **14.** Has your breathing been more difficult after COVID-19? (n=465) | No | 118 (41.5) | 76 (41.8) |
|  | Yes, slightly more difficult | 60 (21.1) | 39 (21.4) |
|  | Yes, more difficult | 85 (29.9) | 56 (30.8) |
|  | Yes, a lot more difficult | 21 (7.4) | 10 (5.5) |
| **15.** Do you still have a cough after COVID-19? (n=466) | No | 201 (70.8) | 132 (72.5) |
|  | Yes, but not bad | 53 (18.7) | 40 (22.0) |
|  | Yes, fairly bad (the cough sometimes interferes with sleep) | 25 (8.8) | 8 (4.4) |
|  | Yes, very bad (the cough interferes with my quality of life) | 5 (1.8) | 2 (1.1) |
| **16.** Do you have pressure wounds? (n=460) | No | 266 (93.7) | 175 (96.2) |
|  | Yes, but a nurse helps me to care for them | 8 (2.8) | 2 (1.1) |
|  | Yes, but I have been told how to care for them | 4 (1.4) | 0 (0) |
|  | Yes, but I have not shown them to anyone | 3 (1.1) | 2 (1.1) |
| **17.** Have you gone back to work, study or previous occupation?  (n=463) | Yes, I have gone back to work/studies | 157 (55.3) | 86 (47.3) |
|  | No, I am off sick | 38 (13.4) | 16 (8.8) |
|  | No, I am unemployed | 14 (4.9) | 9 (4.9) |
|  | No, I have retired | 67 (23.6) | 64 (35.2) |
|  | Other | 6 (2.1) | 6 (3.3) |
| **18.** Have you been back to hospital or contacted healthcare or  rehabilitation due to COVID-19 after being discharged from | Yes, been back to hospital | 27 (9.5) | 17 (9.3) |
| hospital? (n=465) | Yes, been to healthcare center or rehabilitation clinic | 79 (27.8) | 43 (23.6) |
|  | Yes, other | 30 (10.6) | 18 (9.9) |
|  | No | 147 (51.8) | 104 (57.1) |

Data are number of (%).

COVID-19=coronavirus disease 2019.
